# Role of c-Jun N-Terminal Protein Kinase 1/2 (JNK1/2) in Macrophage-Mediated MMP-9 Production in Response to *Moraxella catarrhalis* Lipooligosaccharide (LOS)

**DOI:** 10.1371/journal.pone.0037912

**Published:** 2012-05-24

**Authors:** Ferdaus Hassan, Dabin Ren, Wenhong Zhang, Xin-Xing Gu

**Affiliations:** Vaccine Research Section, National Institute on Deafness and Other Communication Disorders, Rockville, Maryland, United States of America; Massachusetts General Hospital/Harvard Medical School, United States of America

## Abstract

*Moraxella catarrhalis* is a Gram negative bacterium and a leading causative agent of otitis media (OM) in children. Recent reports have provided strong evidence for the presence of high levels of matrix metalloproteinase (MMPs) in effusion fluids from children suffering with OM, however, the precise mechanisms by which MMPs are generated are currently unknown. We hypothesized that MMPs are secreted from macrophages in the presence of *M. catarrhalis* lipooligosaccharide (LOS). In this report, we demonstrate that *in vitro* stimulation of murine macrophage RAW 264.7 cells with LOS leads to secretion of MMP-9 as determined by ELISA and zymogram assays. We have also shown that inhibition of ERK1/2 and p38 kinase completely blocked LOS induced MMP-9 production. In contrast, inhibition of JNK1/2 by the specific inhibitor SP600125 actually increased the level of expression and production of MMP-9 at both mRNA and protein levels, respectively by almost five fold. This latter result was confirmed by knocking down JNK1/2 using siRNA. Similar results have been observed in murine bone marrow derived macrophages *in vitro*. In contrast to and in parallel with the LOS-induced increased levels of MMP-9 in the presence of SP600125, we found a corresponding dose-dependent inhibition of TIMP-1 (tissue inhibitor of matrix metalloproteinase-1) secretion. Results of subsequent *in vitro* studies provided evidence that when JNK1/2 was inhibited prior to stimulation with LOS, it significantly increased both the extent of macrophage cell migration and invasion compared to control cells or cells treated with LOS alone. The results of these studies contribute to an increased understanding of the underlying pathophysiology of OM with effusion in children.

## Introduction

Otitis media (OM) is the most common infectious disease in children and is caused by infection with either virus or bacteria [1]. Until recently, *Streptococcus pneumonia* was recognized as the leading causative pathogen; however, as a consequence of the introduction of the newly developed heptavalent pneumococcal vaccine, the microbiology of OM has changed and nontypeable *Haemophilus influenzae* (NTHi) has become leading the pathogen and *Moraxella catarrhalis (M.cat)* now ranks third (and accounts 15–20% of total bacterial infections) [2], [3]. Most children (80%) experience at least one episode of OM by their third birthday and half suffer multiple episodes of infection [4]. The total health care costs for OM are estimated to be $6 billion annually due to medical cost and lost wages [5]. Similar to other Gram negative bacteria, *M.cat* possesses lipooligosaccharide (LOS), found in the outer membrane of the bacteria. The LOS is structurally distinct from typical lipopolysaccharide (LPS). LOS consists of an oligosaccharide core and associated lipid A without the presence of repeating O-antigen polysaccharide side-chains that are commonly found in LPS [6], [7], [8].

The tissue macrophage is one of the key immune cells usually found at the site of inflammation. In response to pro-inflammatory stimuli such as LOS, these cells produce pro-inflammatory cytokines such as TNF-α, IL-6, nitric oxide etc. Additionally, macrophages also play an important role in tissue damage and tissue remodeling by secreting a variety of different matrix metalloproteinases (MMPs), especially the highly inducible MMP-9 [9]. MMP-9 belongs to a family of zinc- dependent endopeptidase that functions to promote degradation of the extracellular matrix [10]. Recently MMP-2 and MMP-9 have been detected in patients with OM with effusion, as well as in patients with chronic OM with effusion. [11], [12]. Regardless of the status of OM, MMP-9 manifested activity is found in all effusions. Recently we have shown that LOS from *M.cat* has the capacity to activate human monocytes through toll like receptor 4 (TLR4) [13]. However, relatively little is known about *M.cat* LOS induced pathogenicity in OM with effusions. In the same article, we also have reported that *M.cat* LOS selectively increases ICAM-1 expression in human monocytes compared to typical *E.coli* LPS [13] and concluded that these differences might be due to structural differences between LPS and LOS. Here, we report that LOS induced expression and secretion of MMP-9 in murine macrophage like RAW 264.7 cells, as well as in bone marrow derived macrophages (BMMØ). Inhibition of the activity of one of the mitogen activated protein kinase (MAPK) member, JNK1/2, further significantly augmented MMP-9 expression and production at the mRNA and protein level, respectively. Simultaneously, inhibition of JNK1/2 also inhibited tissue inhibitors of matrix metalloproteinase-1 (TIMP-1) secretion, a natural inhibitor of MMP-9 [14]. Finally we showed that increased production of MMP-9 is associated with an increased rate of macrophage cellular migration and invasion.

## Materials and Methods

### Materials

LOS was extracted from a clinical isolate of *M. catarrhalis* strain O35E (Kindly provided by Eric Hansen, University of Texas Southwestern Medical Center, Dallas, TX, USA) as described earlier [15], [16]. The levels of protein and nucleic acid in purified LOS preparation were less than 1%. Lipopolysaccharide from *E.coli* 055:B5 was purchased from Sigma Chemicals (St Louis, MO), JNK1/2 inhibitor SP600125, p38 inhibitor SB202190, ERK1/2 inhibitor U0126, AKT inhibitor LY294002, MMP-2/MMP-9 inhibitor (2R)-2-[(4-Biphenylylsulfonyl)amino]-3-phenylpropionic Acid (BiPS) were all obtained from Calbiochem (San Diego, CA), Recombinant mouse MCSF was purchased from R&D systems (Minneapolis, MN).

### Cell Culture

The murine macrophage like cell line RAW 264.7 was purchased from ATCC (Manassas, VA) and maintained in DMEM medium containing 10% heat inactivated fetal bovine serum (Gibco-BRL, Gaithersburg, MD) and antibiotics at 37°C at humidified condition under 5% CO_2_.

### Isolation of mouse bone marrow derived macrophage

Female C57BL/6J (6 weeks) mice were purchased from Taconic Farm Inc. (Germantown, NY) and maintained in specific pathogen free condition in an animal facility in accordance with National Institutes of Health guidelines. Bone marrow derived macrophages were collected as described earlier [17], [18] with some modification. In brief, mouse tibia and femur were cut at both the end and flushed with RPMI 1640 medium containing 10% FBS. Harvested cells were cultured in the presence of mouse recombinant MCSF at 20 ng/ml for seven days at 37°C under 5% CO_2_. Every two days, old media was replaced with new media containing MCSF at the same concentration. At day seven, adherent macrophages were used for further experiments. All experiments involving mice were performed according to the recommendations in the Guide for the Care and Use of Laboratory Animals of the National Institutes of Health. All protocols were reviewed and approved by institutional review boards at the National Institutes of Health (Permit Number: 1158).

### Zymography

Enzymatic activities of MMP-2 and MMP-9 present in culture supernatants were determined by 10% precast gelatin zymogram gel (Invitrogen, Carlsbad, CA). In brief, cells (1×10^5^/100 µl/well) were cultured in 96 well plate with DMEM containing 0.5% FBS and pretreated with SP600125 or other MAPK inhibitors as indicated in figures, for 1 h before treating with LOS for 18 h. Supernatants were collected and mixed with equal amounts of 2× SDS-sample buffer containing 125 mM Tris-Hcl, 4% SDS, 20% glycerol and 0.001% bromophenol blue in the absence of non reducing agent and incubated at 37°C for 10 minutes. A total of 20 µl of sample was loaded into each well. After electrophoresis, gel was renatured by novex zymogram renaturing buffer (Invitrogen) for 30 min, washed and incubated with novex zymogram developing buffer (Invitrogen) for 18 h at 37°C, stained with IRDye blue protein stain (Licor, Lincoln, NB) for 1 h and destained with water for 2 h. Gels were then visualized and photographs were taken using Odyssey infrared imaging system (Licor Inc, USA).

### Determination of MMP-9 and TIMP-1

The concentrations of MMP-9 and TIMP-1in culture supernatants collected as described above were determined using an enzyme-linked immunosorbent assay (ELISA) kit (R&D Systems, Minneapolis, MN) according to the manufacturer's instruction.

### Real time RT-PCR

Total RNA was extracted from cells using RNeasy mini spin column (Qiagen Sciences, Germantown, MD) according to the manufacturer's instruction. First-strand cDNAs were prepared using TaqMan reverse transcriptase reagents (Applied Biosystem, Foster city, CA) using eppendorf mastercycler (Eppendorf, Hamburg, Germany). The reverse transcribed cDNA samples were amplified and quantified using power SYBR green PCR master mix using Step One real time PCR system (Applied Biosystem) with specific primers according to the manufacturer's protocol. The primer sequences were used as follows: MMP-9, 5-TCGCGTGGATAAGTTCTC-3 (Forward primer), 5-ATGGCAGAAATAGGCTTTGTCTTG-3 (Reverse Primer), Actin, 5-AGCTGCGTTTTACACCCTTT-3, 5-AAGCCATGCCAATGTTGTCT-3. Following amplification, melting curve analysis was performed at temperature between 60°C to 95°C.

### Silencing of JNK1/2 by siRNA

For knock down of JNK1/2 by siRNA, a mixture of four nucleotides (ON-TARGET Plus SMARTpool siRNA) targeting JNK1 and JNK2 were designed and synthesized by Dharmacon (Thermo Fisher Scientific, Lafayette, CO) and used together for transfection in RAW 264.7 cells using Fugene HD transfection reagent (Roche Applied Sciences, Mannheim, Germany) according to the manufacturer's instruction. The sequences used for JNK1 were 5-UAAAUACGCUGGAUAUAGC-3, 5-GUUCUUAUGAAGUGUGUUA-3, 5-GAAGCAAACGUGACAACAA-3, 5-GAAGCAAACGUFACAACAA-3, 5-CAAGAGAUUUGUUAUCCAA-3 and for JNK2 were -5CCGCAGAGUUCAUGAAGAA-3, 5-GCGGAUCUCUGUFFACGAA-3, 5-AAAGAGAGCAUGCGAUUGA-3, 5-GCAUUCAGCUFFUAUCAUU-3. In brief, 2×10^5^ cells were cultured in 24 well plates and different concentrations of siRNA were mixed with Fugene HD and added to the cells. Cells were then incubated for a total of 30 hours. At this point, the cell culture medium was replaced with fresh culture media (contains 0.5% FCS) and cells were then treated with various concentration of LOS for another 18 hours. Supernatants were collected and used to detect the presence of MMP-9 by zymogram and ELISA. At the same time, control siRNA (non target siRNA sequence is 5-UGGUUUACAUGUCGACUAA -3) were used to exclude possible effect of non-specific RNA.

### Martigel invasion and migration assay

*In vitro* martigel invasion assays were performed using BD BioCoat Martigel Invasion Chambers with 8 µm pore size (BD Biosciences, Belford, MA) according to the manufacturer's protocol. In brief, invasion chambers were rehydrated with DMEM medium for 2 h at 37°C. 5×10^5^ cells in DMEM medium containing 10% FBS were added to the upper chamber (Insert) and the wells contained only DMEM medium with 10% FBS without any cells. Cells in the insert were pretreated with SP600125 or MMP-2/MMP-9 inhibitor or both for 1 h followed by stimulation with LOS (100 ng/ml) for 22 h. Noninvasive cells were carefully removed using cotton swab. Cells that were attached at the lower surface of the membrane were fixed and stained with diff-quick stain kit (IMEB Inc, San Marcos, CA). Three different areas were chosen randomly in each well, observed under microscope at ×200 magnification and photographs were taken

### Statistical analysis

Statistical significance was determined by unpaired *t- test* and performed using GraphPad Prism version 5.04 for Windows. (GraphPad Software, La Jolla, California USA). Experimental results are expressed as the mean of triplicates ± standard deviation in at least 3 independent experiments.

## Results

### Increased production of MMP-9 by macrophages in response to LOS

**Figure 1:**
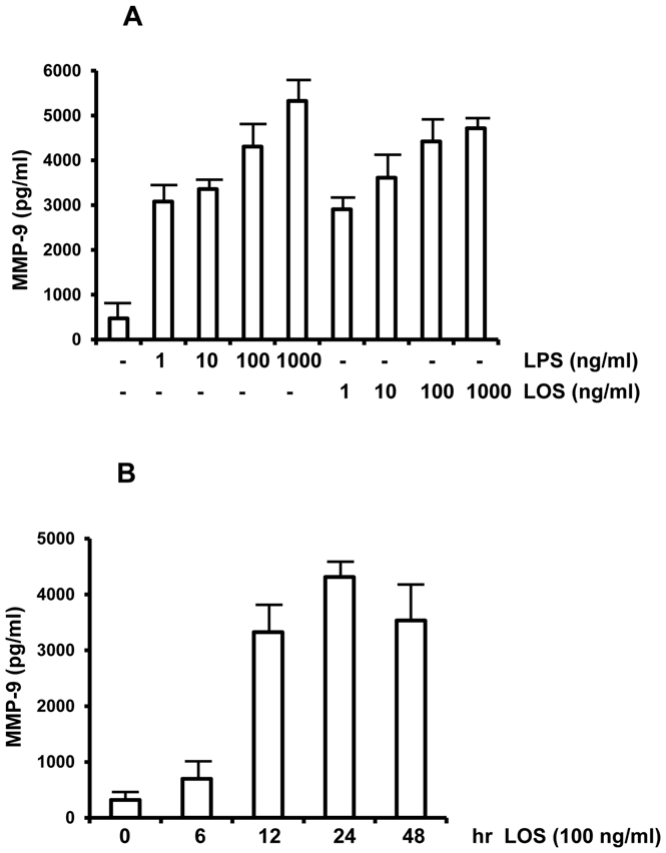
LOS dose-dependently increases MMP-9 secretion in murine macrophage. **A.** RAW 264.7 cells were cultured in 96 well plates at 1×10^5^/100 µl/well concentration and treated with LOS and LPS with different concentration for 18 h and supernatants were collected. Levels of MMP-9 were determined by ELISA. **B.** RAW 264.7 cells were seeded as above and treated with LOS at 100 ng/ml concentration. Supernatants were collected at 6, 12, 24 and 48 hours and MMP-9 was quantified by ELISA. Results are representative of at least three independent experiments.

The concentration and time dependent effect of LOS on MMP-9 production by RAW 264.7 cells was examined. RAW 264.7 cells were treated with various concentrations of LOS, or as a control, with LPS for 18 hours. Production of MMP-9 was examined using MMP-9 specific ELISA kits. Increased levels of MMP-9 were detected in culture supernatants in response to LOS as little as 1 ng/ml compared to untreated cells and reached maximum levels at concentrations of 100–1000 ng/ml of LOS (Fig. 1A). We did not observe a significant difference in response to 100 ng/ml *vs* 1000 ng/ml of LOS in terms of MMP-9 production. Both *E.coli* LPS and *M.cat* LOS stimulated approximately same levels of MMP-9 production at concentration of 100 ng/ml. In the following experiments, therefore, we routinely used LOS at 100 ng/ml concentration unless otherwise stated. The level of MMP-2 remained unchanged at any concentration of LOS as found in both zymogram and MMP-2 specific ELISA (data not shown).

We also evaluated the time course for MMP-9 production by RAW 264.7 cells in response to LOS in RAW 264.7 cells. At 6 hours following LOS treatment, we detected relatively little amount of MMP-9 in culture supernatants but it was readily detected at12 hours, reaching its peak at 24 hours with a slight but probably not significant decrease at 48 hours (Fig. 1B). These data indicates that LOS dose dependently triggers the production and secretion of MMP-9 at 100 ng/ml and 24 hours being the apparent optimal conditions.

### Role of mitogen activated protein kinases (MAPK) in LOS triggered MMP-9 production

**Figure 2:**
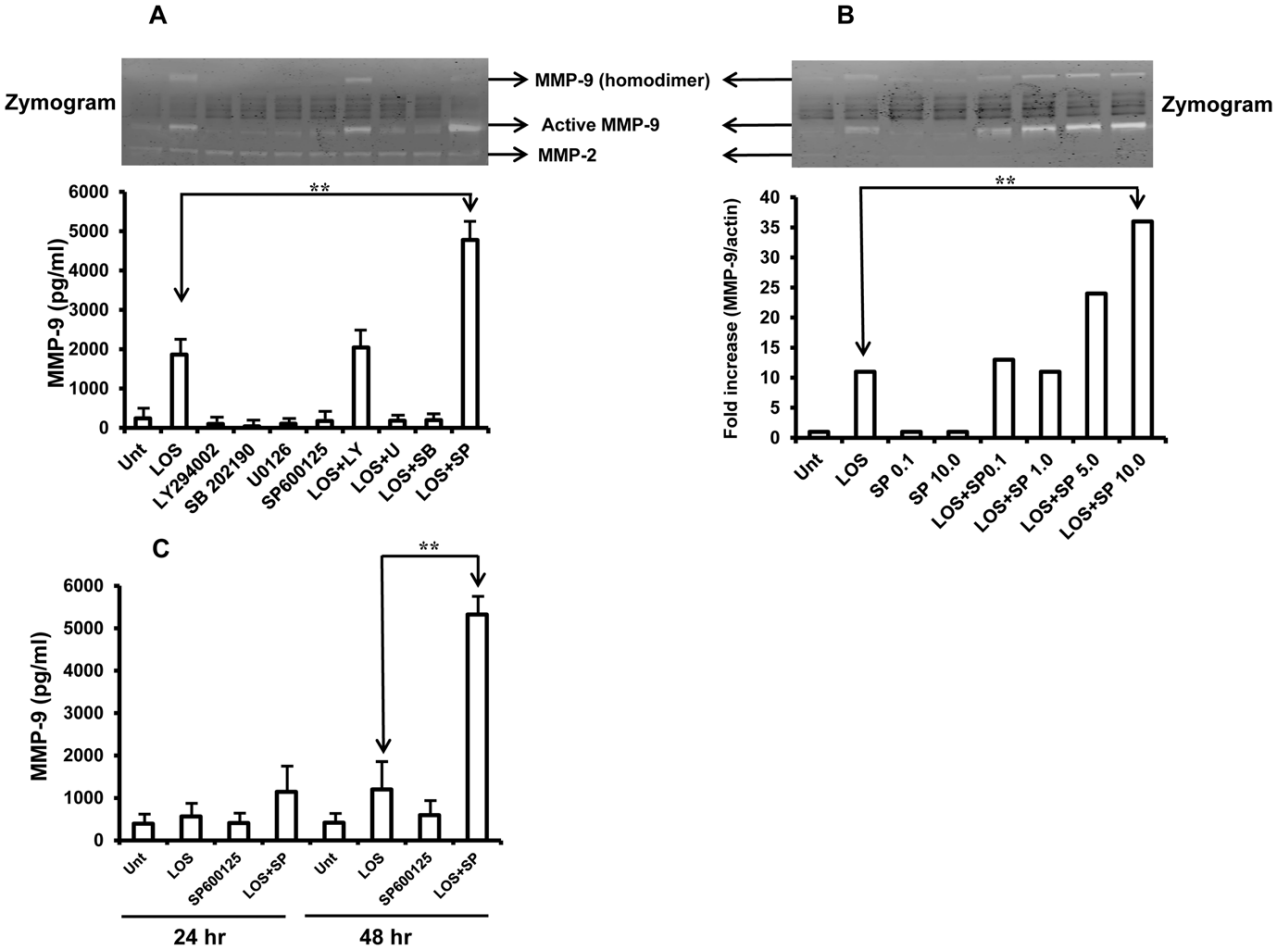
Inhibition of JNK1/2 by SP600125 increases LOS induced MMP-9 production in murine macrophages. **A,** RAW 264.7 cells were seeded in 96 well plate at 1×10^5^/100 µl/well. Cells were pretreated with AKT inhibitor LY 294002 (10 µM) and various MAPK inhibitors (ERK1/2 inhibitor U0126 (10 µM), p38 inhibitor SB202190 (10 µM) and JNK1/2 inhibitor SP600125 (10 µM) for 60 min and followed by LOS (100 ng/ml) treatment for 18 hrs. Supernatants were collected and activity of MMP-9 and MMP-2 were detected by zymogram (upper panel) and levels of MMP-9 were determined by ELISA (lower panel). **B,** RAW 264.7 cells were pretreated with different concentration of JNK1/2 inhibitor (0.1 µM, 1.0 µM, 5.0 µM and 10.0 µM) as indicated in figure followed by LOS treatment as described above. MMP-9 enzymatic activity and MMP-9 gene expression were determined by zymogram (upper panel) and real time-PCR, respectively (lower panel). **C,** Bone marrow derived macrophages were again pretreated with SP600125 (10 µM) for 1 hour followed by LOS treatment (100 ng/ml) for 48 hours. Cell culture supernatants were obtained and levels of MMP-9 were measured using ELISA kit. Typical results of three independent experiments are shown. ** (*p*<0.05, considered significant).

MAPK has been established to play an important role in MMP-9 production in different cell types [19]. The active role of MAPK and/or PI3K in LOS induced MMP-9 production was therefore evaluated in RAW 264.7 cells. Cells were pretreated with a specific inhibitor of the signaling kinases p38 (SB202190), ERK1/2 (U0126), JNK1/2 (SP600125) or PI3K/AKT (LY294002) for 1 h and then treated with LOS (100 ng/ml) for 18 h. The levels of secreted MMP-9 and MMP-2 were then detected using zymography (Fig. 2A, upper panel). In separate studies, we have established that, SP600125 at 10.0 µM concentration completely inhibited LOS induced JNK1/2 activation and also none of the inhibitors when used at these concentrations were toxic (data not shown). LOS, at 100 ng/ml concentration, induced significant production of MMP-9. None of the inhibitors alone stimulated the production of detectable MMP-9. Inhibition of p38 and ERK1/2 resulted in complete abolishment of LOS-induced MMP-9 production. Somewhat surprisingly, inhibition of JNK1/2 by SP600125 resulted in augmented secretion of MMP-9. To confirm these findings, we purchased the same inhibitor from a second commercial source (Biomol Inc, Plymouth Meeting, PA) and obtained equivalent results. Inhibition of the AKT pathway had no detectable effect on MMP-9 secretion. In addition, none of the inhibitors tested alone or in combination had any detectable effect on LOS-induced MMP-2 secretion. We also monitored the levels of MMP-9 in culture supernatants using MMP-9 specific ELISA (R&D Inc, USA) and observed significant increase of MMP-9 secretion by JNK1/2 inhibition compared to LOS alone (*p*<0.05) (Fig. 2A, lower panel).

We then investigated whether or not SP600125 effects the gene expression of MMP-9. Cells were pre-treated with various concentrations of SP600125 with or without subsequent stimulation with LOS for 18 hours and total RNA were then extracted from the cells. A total 200 ng of RNA was converted to cDNA and levels of gene expression of MMP-9 were determined by real time PCR (Fig. 2B, lower panel). The levels of gene expression of MMP-9 were found to be significantly increased by almost ten folds with LOS treatment compared to untreated cells. Importantly, however, in cells that were treated with both SP600125 and LOS, levels of MMP-9 mRNA expression were dose-dependently increased by twenty folds and four folds compared to untreated cells and LOS treated cells, respectively. In similar experiments, culture supernatants were collected and enzymatic activity of MMP-9 was detected by zymogram (Fig. 2B, upper panel). Together, the data in this figure provide strong support for the conclusion that the specific JNK1/2 inhibitor along with LOS dose-dependently increases expression of MMP-9 at both the mRNA and protein level.

Next, we examined the extents to which the results so far obtained with the RAW 264.7 cell line would be applicable to primary mouse macrophage. We therefore carried out studies to assess the effect of SP600125 and LOS on MMP-9 production in bone marrow derived macrophages (BMMØ) *in vitro*. BMMØ generated as described in Materials and Methods were pretreated with medium alone or with SP600125 at 10 µM concentrations for 1 hour followed by LOS treatment for 24–48 hours and levels of MMP-9 were quantified in culture supernatants (Fig. 2C). There were no significant levels of MMP-9 detected until 24 h in response to LOS compared to untreated cells. However, inclusion of SP600125 again resulted in increased levels of MMP-9 secretion within 24 hours compared to LOS alone. Furthermore, relatively robust levels of MMP-9 secretion were observed at 48 hours when cells were treated with the JNK1/2 inhibitor and LOS compared to LOS alone (*p*<0.05). It should be noted that, unlike RAW 264.7 cell responses, we observed relatively high amounts of MMP-9, even in untreated samples. This is perhaps due to the fact that primary cells were previously cultured in the presence of M-CSF for seven days, which might lead to secretion of MMP-9 even in the absence of LOS treatment. These data further confirm our earlier observations and together these data provide strong evidence for negative regulation of MMP-9 production in murine macrophage in response to LOS by JNK1/2.

### Knock down of JNK1/2 by siRNA increases MMP-9 production

**Figure 3:**
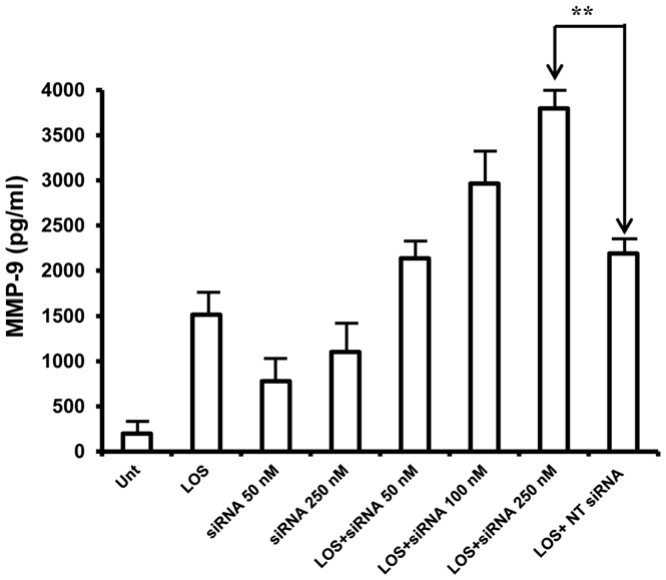
Knock down of JNK1/2 by siRNA enhances secretion of MMP-9 in RAW 264.7 cells. RAW 264.7 cells were transfected with JNK1/2 specific siRNA at various concentration for 30 h and then treated with LOS (100 ng/ml) for another 18 h. Supernatants were collected and levels of MMP-9 were quantitated by ELISA. One representative data from total of three experiments is shown. NT siRNA, non-target siRNA. **(*p*<0.05, considered significant).

Since SP600125 is a pharmacological inhibitor with preferred specificity for JNK1/2, we cannot exclude the possibility of non-specific inhibitory effects on other signaling pathway [20]. To confirm our observation, therefore, we adopted an additional approach by knocking down JNK1/2 expression using siRNA and then treating with LOS and measuring levels of MMP-9 secretion by ELISA as previously described (Fig. 3). In separate studies, we found that almost 70% of total JNK1/2 expression was inhibited by specific siRNA when used at 250 nM concentration compared to non-target siRNA (data not shown). RAW 264.7 cells that were transfected with siRNA against JNK1/2 and subsequently treated with LOS, secreted significantly more MMP-9 than cells that were transfected with non-target siRNA (NT siRNA) (*p*<0.05) and similarly treated with LOS. The extent of increased production of MMP-9 correlated approximately with the siRNA concentrations used to knock down JNK1/2 expression. Cells that were transfected with either siRNA for JNK1/2 alone, or with control siRNA secreted some detectable levels of MMP-9. This is perhaps due to the effect of transfection reagent which is based on cationic lipid or could be non-specific effect of siRNA itself. We assume the later since siRNA without LOS treatment dose-dependently increased secretion of MMP-9 however, the levels of MMP-9 observed were significantly lower compared to LOS alone. The results of this study further confirm a key role for JNK1/2 in MMP-9 secretion in murine macrophages stimulated with LOS. However, silencing JNK1/2 had no effect on MMP-2 production (data not shown) providing additional supporting evidence that JNK1/2 specifically and negatively regulates MMP-9 secretion but not MMP-2.

### Inhibition of JNK1/2 results in decreased TIMP-1 secretion in macrophages to LOS

**Figure 4:**
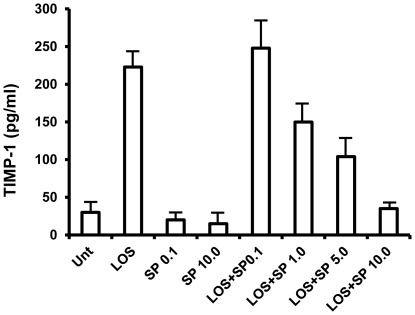
SP600125 selectively inhibits LOS-induced secretion of tissue inhibitor of matrix metalloproteinase-1 (TIMP-1) in macrophages. RAW 264.7 cells were seeded in a 96 well tissue culture plate as above and pretreated with SP600125 at different concentration for 1 h followed by LOS (100 ng/ml) treatment for 18 hrs. Supernatants were collected and levels of TIMP-1 were determined by ELISA. Results are representative of at least two independent experiments.

It is well documented that tissue inhibitors of matrix metalloproteinaeses-1 (TIMP-1) function as a natural inhibitor of MMPs [14]. We therefore investigated the potential role of TIMP-1 in the observed JNK1/2 regulated MMP-9 production in macrophages followed by LOS stimulation. RAW 264.7 cells were, as described above, pretreated with SP600125 for 1 hour followed by LOS treatment for 18 hours and supernatants were collected to quantitate levels of TIMP-1 secreted in culture media by ELISA. Inhibition of JNK1/2 by SP600125 dose dependently decreased secretion of TIMP-1 in mouse macrophage 264.7 cells compared to LOS alone (Fig. 4). The effects of SP600125 were detected with as little as 100 nM of inhibitor as assessed by TIMP-1 secretion and secretion was completely inhibited at 10.0 µM. These data suggest a possible role of TIMP-1 in JNK1/2 regulated augmented production of MMP-9 in response to LOS.

### Inhibition of JNK1/2 increases cellular invasion and migration

**Figure 5:**
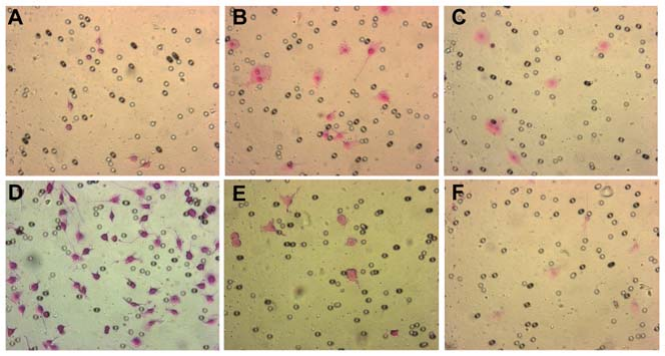
LOS induced MMP-9 is the key molecule responsible for macrophage invasion. RAW 264.7 cells were cultured in martigel invasion chamber (8.0 µM pore size) and treated with LOS and SP600125 as described in materials and methods. After 18 h of incubation, upper surface cells were removed and cells migrated to lower surface were stained with H&E and observed under microscope. A, untreated, B, LOS (100 ng/ml), C, SP600125 (10 µM), D, LOS+SP600125, E, LOS+MMP-9 inhibitor, F, LOS+SP600125+MMP-9 inhibitor. Magnification ×200. Typical results of three independent experiments are shown.

Accumulating evidence suggests a potential key role of MMPs in cellular invasion and migration [21]. Since JNK1/2 inhibition results in an augmented MMP-9 secretion, we hypothesized that inhibition of JNK1/2 activity will also lead to increase invasion and migration. To test this hypothesis, we cultured RAW 264.7 cells in a martigel invasion assay chamber with 8.0 µm pore size and treated the cells with LOS in the presence or absence of SP600125 or MMP-9 inhibitor for 24 hours. Few cells were found to be able to invade the martigel and had migrated to the lower chamber in response to LOS treatment alone compared to untreated cells. There were no cells either invaded or migrated to lower chamber that could be detected when treated with SP600125 alone. However, significantly greater number of cells were found in the lower chamber when cells were treated with both SP600125 and LOS compared to LOS alone (Fig. 5 A–D). To confirm the role of MMP-9 in cellular migration and invasion, we pretreated the cells with an MMP-2/9 inhibitor followed by SP600125 and finally with LOS. As expected, there were almost no cells detected in the lower chamber of the plate (Fig. 5E), thereby helping to confirm our hypothesis that LOS induced MMP-9 is one of the primary macrophage products contributing to macrophage invasion and migration. Cells that were treated with only LOS and MMP-2/9 inhibitor had no detectable effect on either invasion or migration (Fig. 5F).

## Discussion

Acute otitis media (AOM) or otitis media with effusions can be broadly characterized by the accumulation of fluid and pain in the middle ear space with or without any sign or symptoms of infection [22]. Irrespective of sign or symptoms, sustained infection can bring potential pathogen such as bacteria or virus from epipharynx to the middle ear. Pathogen or its byproduct can stimulate local mucosal cells attracting immune-effector cells, leading to an inflammatory response that is largely responsible for the clinical manifestations which occur in the middle ear [23] Initially, the tympanic membrane remains intact, however, as the disease progresses, the structure of tympanic membrane changes due to degradation of structural macromolecules of connective tissues [24], [25]. These changes in the tympanic membrane impact the overall hearing function. There are several mechanisms proposed for this degradation and it is now well known that MMPs, tissue plasmin and other proteases play a critical role for the degradation of tympanic membrane [26]. Recently it has been found that cultured tympanic membrane expresses MMPs in response to bacterial inflammatory mediators including endotoxins and high level of MMPs were also found in acute post tympanostomy otorrhea (APTO) in human [27], [28]. These evidences strongly suggest that there is a direct link between infection and expression and secretion of MMPs which, if over-produced, can be destructive to tissue and may contribute to the pathogenicity of OM. LOS is one of the key virulence factors associated with *M.Cat* and structurally quite distinct from typical LPS structure [29], [6], [8]. Despite these structural differences, both LOS and LPS have the similar capacity of cellular activation; at least in terms of MMP-9 production in murine macrophages (Fig. 1).

Mitogen activated protein kinases (MAPK) are protein Ser/Thr kinases that convert extracellular stimuli to intracellular signaling [30] and regulates wide range of cellular function such as gene expression, mitosis, metabolism, motility, survival, apoptosis and differentiation. Although there are total of 14 MAPKs so far have been identified, however, three of them are most widely studied (i) extracellular signal regulated kinase 1/2 (ERK1/2), (ii) p38 MAP kinase and (iii) c-Jun N-terminal kinase 1/2 (JNK1/2). Here, in this report, using specific inhibitors of various MAP kinases, we observed that p38 and ERK1/2 positively regulate the secretion of MMP-9 while PI3 kinase had no effect on MMP-9 secretion (Fig. 2A). Similar results have been reported earlier that showed activation of p38 was necessary in LPS induced MMP-9 secretion in murine macrophage [31]. In our study, inhibition of JNK1/2 not only increased MMP-9 secretion in murine cell line but also from bone marrow derived macrophages (Fig. 2C) suggesting that augmented MMP-9 secretion is not unique to RAW 264.7 cell but also applicable to mouse primary cells.

MMPs are produced in an inactive form and their activities are regulated by endogenous inhibitor such as tissue inhibitors of metalloproteinases (TIMPs) and disruption of the MMP-TIMP balance can result in a number of pathogenic processes including tumor invasion, metastasis, angiogenesis and wound healing [14]. Our data indicates that LOS augmented levels of secretion of both MMP-9 and TIMP-1, this is perhaps due to the fact that synthesis of MMP-9 and TIMP-1 are under the control of same transcription factors such as AP-1, CREB and NF-κB and all these factors can be activated by LOS [32]. Since, inhibition of JNK1/2 by SP600125 inhibited LOS induced TIMP-1 secretion, it is possible, at least partially, that augmented production of MMP-9 is due to inhibition of TIMP-1 secretion. The other possible mechanism might be at transcription level since inhibition of JNK1/2 also increased levels of MMP-9 gene expression (Fig. 2). The exact mechanism on how SP600125 augments MMP-9 secretion needs further investigation. It is now well documented that MMPs are responsible for tissue invasion and angiogenesis. MMP-2 and MMP-9 are not only found in OM with effusion but also in pediatric patients with cholesteatoma, a chronic stage of otitis media [11], [12], [33]. Consistent with other clinical studies, we found strong correlation between MMP-9 secretion and transwell migration and invasion (Fig. 5) by murine macrophage in response to LOS together with JNK1/2 inhibition.

In summary, in this report, we have provided strong evidences that LOS, a key virulence factor of *M.cat*, induced MMP-9 but not MMP-2 secretion in murine macrophage RAW 264.7 cells. Inhibition of JNK1/2 by SP600125 further augmented LOS induced MMP-9 secretion in both RAW 264.7 cells and bone marrow derived primary macrophages which in turn lead to increased cellular invasion and migration. In past several years, pharmaceutical companies have been developing small molecules targeting JNK1/2 signaling pathway to treat various diseases such as cancer, Alzheimer, ischemia-reperfusion injury and wound healing [34]. However, contradictory results have been reported in I/R injury animal model using SP600125. Three independent groups reported that SP600125 reduces I/R injury in the brain and lungs and protects hepatocytes from TNF-α induced apoptosis [35], [36], [37] while lee *et. al.* reported harmful effect of SP600125, showing that administration of this compound increases serum ALT level 24 hours after reperfusion with more severe parenchymal destruction, leukocyte infiltration and MMP-9 secretion [38]. More recently, Allan F. Ryan and his group found that SP600125 significantly inhibited mucosal hyperplasia during *in vivo* bacterial otitis media in guinea pigs [39]. More studies are needed to develop effective JNK1/2 inhibitor as a therapeutic target to treat various diseases including OM.
